# The Short-Term Impact of Educational Programs on Knowledge and Attitudes Regarding Antimicrobial Stewardship among Veterinary Students in Serbia

**DOI:** 10.3390/ani14182736

**Published:** 2024-09-21

**Authors:** Zorana Kovačević, Nikola Čudina, Marko Pećin, Marko Samardžija, Marko Pajić, Selma Pintarić, Ivan Vlahek, Zoran Ružić, Vuk Vračar, Ivan Galić, Olga Horvat

**Affiliations:** 1 Department of Veterinary Medicine, Faculty of Agriculture, University of Novi Sad, Trg Dositeja Obradovica 8, 21000 Novi Sad, Serbia; zorana.kovacevic@polj.edu.rs (Z.K.); zoran.ruzic@polj.uns.ac.rs (Z.R.); vuk.vracar@polj.uns.ac.rs (V.V.); ivan.galic@polj.uns.ac.rs (I.G.); 2 Department of Pharmacology and Toxicology, Faculty of Veterinary Medicine, University of Zagreb, Heinzelova 55, 10000 Zagreb, Croatia; ncudina@vef.unizg.hr; 3 Clinic for Surgery, Orthopedics and Ophthalmology, Faculty of Veterinary Medicine, University of Zagreb, Heinzelova 55, 10000 Zagreb, Croatia; 4 Clinic for Reproduction and Obstetrics, Faculty of Veterinary Medicine, University of Zagreb, Heinzelova 55, 10000 Zagreb, Croatia; smarko@vef.unizg.hr; 5 Department for Epizootiology, Clinical Diagnostic, Pathology and DDD, Scientific Veterinary Institute “Novi Sad”, Rumenački Put 20, 21000 Novi Sad, Serbia; markopajic@niv.ns.ac.rs; 6 Department of Microbiology and Infectious Diseases with Clinic, Faculty of Veterinary Medicine, University of Zagreb, Heinzelova 55, 10000 Zagreb, Croatia; 7 Department of Animal Breeding and Livestock Production, Faculty of Veterinary Medicine, University of Zagreb, Heinzelova 55, 10000 Zagreb, Croatia; ivlahek@vef.unizg.hr; 8 Department of Pharmacology and Toxicology, Faculty of Medicine, University of Novi Sad, Hajduk Veljkova 3, 21000 Novi Sad, Serbia; olga.horvat@mf.uns.ac.rs

**Keywords:** antimicrobial resistance, antimicrobial stewardship, veterinary education, knowledge assessment, attitudes

## Abstract

**Simple Summary:**

The resistance to antimicrobial drugs is a growing concern in both human and veterinary medicine. Adequate and responsible use of antimicrobial drugs is crucial in combating this threat. Identifying the gaps in knowledge and attitudes, and ensuring the appropriate education of future doctors of veterinary medicine on this matter, are prerequisites for ensuring the efficacy of these drugs in future. This study evaluated the impact of an additional educational program in the form of a symposium on the knowledge and attitudes of veterinary students in Serbia regarding the responsible use of antimicrobial drugs. When compared with their baseline knowledge and attitudes, attending the educational program improved the knowledge and attitudes of Serbian veterinary students which shows the benefits and importance of integrating targeted educational programs into veterinary curricula.

**Abstract:**

Effective antimicrobial stewardship (AMS) is crucial for combating the rise of antimicrobial resistance (AMR), particularly in veterinary medicine. Educational programs targeting veterinary students can play a significant role in shaping their knowledge and attitudes toward antimicrobial use and stewardship. This study aimed to evaluate the impact of educational programs on the knowledge and attitudes regarding AMS among veterinary students in Serbia. A structured educational program on AMS was implemented for veterinary students at the Faculty of Agriculture, University of Novi Sad. Pre- and post-symposium assessments were conducted to measure changes in students’ knowledge and attitudes. The study employed a mixed-methods approach, including surveys and focus groups, to gather quantitative and qualitative data. The study resulted in significant short-term improvements in students’ knowledge of AMS principles and their attitudes toward responsible antimicrobial use. Participants demonstrated a better understanding of the mechanisms of resistance and the importance of adhering to stewardship guidelines. Qualitative feedback indicated increased awareness of the consequences of inappropriate antimicrobial use and a stronger commitment to applying stewardship practices in their future careers. The educational programs effectively enhanced immediate veterinary students’ knowledge and attitudes regarding AMS. These findings underscore the importance of integrating targeted educational programs into veterinary curricula to promote responsible antimicrobial use and combat resistance in veterinary practice.

## 1. Introduction

Antimicrobial resistance (AMR) has become one of the major global health concerns [1,2]. By the year 2050, AMR is projected to result in approximately 10 million annual deaths worldwide, with an estimated economic burden of US$ 100 trillion [3,4]. The development of increasingly complex databases and surveillance systems both in human and veterinary medicine shows that high-quality research, data, and analysis are crucial for guiding new actions against AMR and supporting decision-makers in enhancing current measures [5]. The essential strategy for combating this threat, antimicrobial stewardship (AMS) heavily relies on the education of all current and future drug prescribers, including human and veterinary doctors. Although Day One Competencies for newly graduated veterinarians include understanding animal disease treatment, prevention, and diagnosis, medicine legislations and guidelines regarding the use of antimicrobial drugs, and disease prevention including promotion of health and welfare, there is a reported lack of such expertise [6]. Furthermore, self-efficacy, defined as the belief that one can perform the individual steps that comprise a task, in choosing the correct antimicrobial treatment is associated with factors including empirical selection and dosing of antimicrobials, identification of trustworthy resources and resistance to pressure to prescribe, and knowledge of when antimicrobials are needed. Besides, although students do show a great increase in self-efficacy by their fourth year of veterinary studies, involvement in the clinical rotations does not show a significant effect [7].

Understanding the knowledge and perceptions of students who will be prospective antimicrobial prescribers is equally important for guiding the development of curricula that foster best practices in AMS. However, there are a limited number of studies exploring the perceptions of veterinary students regarding AMS globally. A national cross-sectional survey administered across veterinary schools in Nigeria showed a lack of knowledge and AMR awareness with a minority of students being aware of AMS and global efforts in fighting AMR. Moreover, the majority of students expressed a need for more education on clinical use and prescriptions of antimicrobials before graduation which shows the will in students for improvement. Still, final-year students showed better knowledge and understanding of AMR than preclinical students [8]. An inter-university study in Poland showed a similar trend with knowledge about antibiotics and AMR increasing with successive years of veterinary education. Most students did perceive AMR as a significant problem, but only one third of respondents reported awareness of the One Health approach. Only a simple majority of the students believed AMR to be a global problem [9]. In a questionnaire-based study performed in veterinary schools in Serbia and Croatia, students showed modest awareness of veterinary medicine’s contribution to overall AMR and limited knowledge about first-line antibiotic treatment choices. Surprisingly, students with perceived insufficient knowledge of antimicrobials in self-assessment achieved better results in scenario-based knowledge evaluation. The majority of students indicated that the amount of time spent teaching the pharmacology of antimicrobials within their curriculum is not enough [10]. Similar dissatisfactory results were recorded in the studies conducted among veterinary students in Australia, South Africa, and Bangladesh [11,12,13]. Therefore, more training and education are needed in AMS for better performance in reducing resistance patterns [14].

Both human and veterinary medicine educational efforts have been put mostly at the post-graduate level. Moreover, interventional educations are now recommended to start in the early undergraduate phases of studies [15]. To the best of our knowledge, this is the first study conducted among veterinary students where the impact of educational interventions on AMS was measured. So far, this type of study has been conducted among healthcare providers, schoolchildren parents, high school students, and pharmacists with promising results [2,16,17,18]. Antibiotic management education for health professionals including veterinary students provides basic knowledge and facilities to support optimal prescription. Education using passive techniques is quite effective for increasing prescriber knowledge, while those using active techniques are more effective for changing prescribing behavior. Additionally, effective educational techniques are recommended to increase knowledge about antibiotics and improve their use [19].

There is a lack of studies assessing the effect of educational interventions on the knowledge and attitudes of veterinary students toward AMR and AMS. Hence, the present study aimed to assess the impact of an educational symposium on improving the knowledge and attitudes of veterinary students towards AMS and enhancing their ability to appropriately select the correct antimicrobial therapy.

## 2. Materials and Methods

### 2.1. Study Design and Participants 

This is an experimental study with a one-group pretest–posttest design conducted in November 2023 to determine the impact of education in increasing veterinary students’ immediate knowledge and attitudes towards AMS. Veterinary students from the Department of Veterinary Medicine, Faculty of Agriculture, University of Novi Sad, Serbia attended the 3rd Symposium for students of veterinary medicine with international participation entitled “Antimicrobial resistance—the present challenge, the future threat”, organized to mark World Antimicrobial Awareness Week given by the World Health Organization. The symposium was held in Novi Sad, Serbia. Students of veterinary medicine at the university who were willing to participate were included in the study. A total of 246 students from the 2nd to the 6th year at the Department of Veterinary Medicine during the 2023/24 academic year were invited to participate in the symposium. Since first-year students only learn basic sciences and some preclinical subjects, we considered the scope of interventional training to be above their level of training and therefore excluded them from the study. Given the major differences in exposure to clinical subjects and experience, 2nd and 3rd year students, and 4th, 5th and 6th year students, were dichotomized as preclinical and clinical students, respectively. The questionnaire-based survey was administered via an online format using a QR code before and after attending the symposium. Veterinary students were requested to complete an online pretest questionnaire, which was administered via an online format using a QR code before the beginning of the two-day symposium; they were given 20 min to finish the form before the link was disabled. After attending the first day of the symposium, the same subject group was provided with the same questionnaire to assess the impact of the symposium on boosting knowledge and attitudes concerning AMS. 

### 2.2. Ethical Approval 

The study was approved by the Ethical Committee of the Faculty of Agriculture, University of Novi Sad (1000/0102 approval number 1812/2/1) before the research was conducted. The students were asked to sign an electronic information agreement and were informed that their participation was voluntary, that they could abandon completing the survey at any stage, and that their responses would be kept confidential and anonymous. 

### 2.3. The Educational Programs

A two-day symposium was conducted at the Faculty of Agriculture, University of Novi Sad from 16 November to 17 November 2023. It was carried out via theoretical lectures and workshops prepared and presented by the teachers from the Department of Veterinary Medicine, Faculty of Agriculture, University of Novi Sad, Serbia, and the Faculty of Veterinary Medicine, University of Zagreb, Croatia, as well as by a researcher from the Scientific Veterinary Institute ‘Novi Sad’, Novi Sad, Serbia. The symposium included three theoretical lectures related to the ways of the spread of resistance and examples of multidrug-resistant bacteria in dogs and cats, surgical site infections, and optimization of antibiotic use in veterinary medicine. Additionally, the symposium included three workshops: the first, entitled “Pregnancy in Bitches—When and Whether to Use Antibiotics?”; the second, entitled “Antibiotic-Free Poultry—Myth or Reality?”; and the third, entitled “Post-Weaning Diarrhea in Piglets—Challenges and Opportunities”. 

### 2.4. Instrument

The questionnaire (Supplementary File S1) was created by a combination of questions from the surveys with a similar goal as performed by Sarwar et al. [20] and Tahoon et al. [2], with the modifications necessary to ensure correct answers to questions and claims and to be more suitable for prospective veterinarians who will be able to prescribe and treat animals with antibiotics. The necessary modifications were made by the investigating team consisting of doctors of veterinary medicine, medical doctors, pharmacists, and statisticians.

The instrument for data collection used a four-part questionnaire. The first section consisted of five questions regarding the sociodemographic characteristics of the veterinary students (gender, age, field of interest in veterinary medicine, year of study, and previous participation in similar symposiums/workshops). In the second section, the respondents were presented with eighteen statements with five different levels of agreement (completely agree, somewhat agree, neither agree nor disagree, somewhat disagree, and completely disagree). The statements were used to assess the knowledge of students regarding antimicrobial use and AMR. The third section had eleven questions about students’ specific knowledge of the rational use of antimicrobials in veterinary practice. The goal was to assess whether the students were familiar with this issue in practice. Finally, in the fourth section, the students were presented with fourteen statements with also five different levels of agreement to assess their attitudes regarding the use of antimicrobials and AMR. 

Materials in the form of printed PowerPoint presentations of each lecture and workshop were provided to the students by the lecturers at the beginning of the symposium. The QR code leading to Questionnaire 1 was provided at the beginning of the materials, while the QR code leading to Questionnaire 2 was provided at the end of the materials.

### 2.5. Data Analysis 

Descriptive and comparative statistical data analysis was performed with TIBCO Statistica v 14.1.0.8. (TIBCO Software Inc., Palo Alto, CA, USA). All analyzed data were ordinal, so median, lower, and upper quartile were used as measures of central tendency and variability, respectively. Nominal variables were expressed as percentages. The significance of differences in responses between preclinical and clinical students before and after the symposium and in the students of the same age before and after the workshop were analyzed with the Mann–Whitney U test. Differences in knowledge (frequency of correct and incorrect answers/opinions) were tested using the Chi-square test. All statistical hypotheses were tested at a level of statistical significance of 0.05.

## 3. Results

### 3.1. Sociodemographic and Academic Characteristics

A total of 88 students took part in the study resulting in a response rate of 35.77%. Most of the participants were female (n = 65, 73.86%). Respondents reported their current year of veterinary studies with most of them being fourth-year students (n = 27, 30.69%) and only nine second-year students (10.23%). Since veterinary studies in Serbia last for 6 years, reported years of studies were further divided into two phases. Accordingly, the majority of participants were in their second phase (i.e., clinical) (n = 59, 67.05%) and the rest of them were in their first phase of veterinary studies (preclinical) (n = 29, 32.95%). When asked about their main area of interest, about a third (35.23%) of respondents reported small companion animals. A similar number of students showed equal interest in all areas (n = 26, 29.55%), but only six (6.82%) students identified public health, government, industry, and research as their main interests. More than half of the participants (61.36%) never previously engaged in activities or conferences related to this topic. The socio-demographic and academic characteristics of the respondents are shown in Table 1. 

### 3.2. Knowledge about Antimicrobial Use and AMR

In the second part of the questionnaire, the students were given a list of statements that they had to evaluate for truthfulness using a Likert scale to estimate the difference in their general knowledge regarding antimicrobial use and AMR prior to and after the symposium. The complete list of all questions with the respective “desirable” answers is provided in the Supplementary File S1. For the statement “Resistance cannot be transferred between different bacterial species” all students showed a significant improvement in answers with the median answer changing from “Neutral” to “I agree” in preclinical students (*p* = 0.008). Clinical students showed more certainty in their correct answers after the symposium (*p* < 0.001), which was inferred from the shift in the interquartile range towards desirable answers. Preclinical students showed a positive significant change in their evaluation of the statement “Regular use of antibiotics can prevent the spread of resistant bacterial isolates” with the median answer changing from “I disagree” to “I strongly disagree” after education (*p* = 0.004). Preclinical students also showed advancement in their assessment of the statement “Vertical gene transfer in bacteria does not affect the spread of resistance” (*p* = 0.032). Both preclinical and clinical students presented significant enhancement when answering the statement “Environmental bacteria that are not pathogenic to animals can serve as donors of resistance genes” as preclinical students displayed greater certainty in their assessment (*p* = 0.002) and clinical students changed their median answer from “Neutral” to “I agree” (*p* = 0.001). The highly significant difference in answers is evident in answers to the statement “Horizontal transfer of resistance genes can take place between two unrelated bacteria” where both preclinical and clinical students’ median answer changed from “Neutral” to “I agree” (*p* < 0.001). A similar change is observed in answers to the statement “Bacterial resistance to antimicrobial drugs can arise spontaneously without prior contact with the antibiotic”. Following the educational programs, preclinical students expressed greater assurance in their positive assessment of the statement “The same antibiotic resistance gene can be present in two different bacterial species” (*p* = 0.034). Clinical students’ judgment of the latter statement also improved with the median answer changing from “Neutral” to “I agree” after the symposium (*p* < 0.001). All median answers and interquartile ranges before and after education are shown in Table 2.

There were differences noted between the knowledge of preclinical and clinical students both before and after the symposium. After the symposium, compared to preclinical students, clinical students exhibited greater certainty in their assessment of the statements “Antibiotics are useful in the treatment of viral infections (e.g., influenza)” (*p* = 0.002), “By killing the body’s physiological flora, antibiotics allow the development of secondary infections” (*p* = 0.038), and “Antibiotics can kill the physiological flora of the body” (*p* = 0.001) with the same observation before the symposium for the latter statement (*p* = 0.015). 

Significant differences were displayed before the symposium for the statement “Small doses of antibiotics in bacteria cannot stimulate the development of resistance” with clinical students exhibiting a stronger sense of certainty in their answers (*p* = 0.013) whereas preclinical students more appropriately assessed the statement “The same antibiotic resistance gene can be present in two different bacterial species” before the symposium (*p* = 0.008). The list of all the differences between preclinical and clinical students’ assessments is displayed in the Supplementary File S2. 

**Figure 1:**
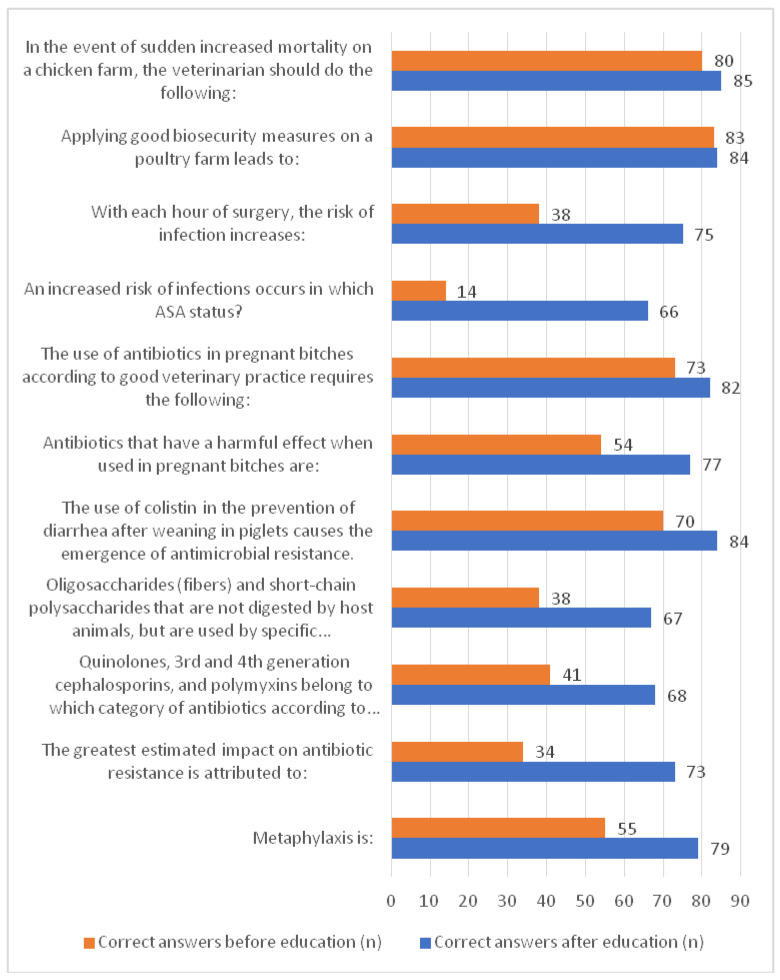
Frequency of correct and wrong answers to multiple-choice questions related to knowledge on rational use of antimicrobials in veterinary practice regarding practical issues.

The third part of the questionnaire consisted of multiple-choice questions focused on determining the students’ specific knowledge of the rational use of antimicrobials in veterinary practice regarding practical issues. Correct answers are presented in Table 3, while the number of correct answers from all respondents before and after the symposium is given in Figure 1. 

The smallest difference between the knowledge of all students before and after the knowledge is observed in the question “Applying good biosecurity measures on a poultry farm leads to:” where the correct answer “Reduced use of antimicrobial drugs at the farm” was chosen by 83 (94.32%) students before and 84 (95.45%) students after the symposium. In contrast, the correct answer “ASA III” to the question “An increased risk of infections occurs in which ASA (American Society of Anesthesiologists) status?” was provided only by 14 (15.91%) students before the symposium, which drastically improved to 66 (75%) correct answers after.

As the answers to the Likert scale-based questions of the second part of the questionnaire were later dichotomized into wrong and correct answers, they were analyzed together with the answers to the multiple-choice questions of the third part of the questionnaire which presented a highly significant difference between the gross knowledge of all the attendants before and after the symposium. The quantities of both correct and wrong answers for all questions regarding the knowledge about antimicrobial use and AMR, as well as the knowledge on rational use of antimicrobials in veterinary practice regarding practical issues, are shown in Table 4.

### 3.3. Attitudes on the Use of Antimicrobial Drugs and AMR

In the fourth part of the questionnaire, the students expressed their level of agreement with a list of statements regarding the use of antimicrobial drugs and AMR. Both preclinical (*p* = 0.001) and clinical (*p* = 0.003) students confirmed increased familiarity with the concept of AMS after the symposium. Students also had an elevated perception of their knowledge about AMR and the rational use of antimicrobials after the symposium as fewer students considered it to be insufficient. Highly significant changes (*p* < 0.001) were observed for both preclinical and clinical students for the questions “I am familiar with the ABCD categorization of antibiotics in veterinary medicine” and “I always find the subtherapeutic use of antibiotics undesirable”. On the other hand, only clinical students showed improvement in median answers for the questions “Veterinarians know enough about the correct use of antibiotics” (*p* = 0.028), “Treating animals with the wrong antibiotics will cause AMR in humans” (*p* = 0.003), and “Broad-spectrum antibiotics are a justified choice for the treatment of all bacterial infections” (*p* = 0.033). All statements and attitude medians with respective interquartile ranges and *p*-Values can be found in Table 5.

Whereas differences were noted between the attitudes and knowledge of preclinical and clinical students, significant distinctions in their attitudes were perceived only in the statement “I am familiar with the ABCD categorization of antibiotics in veterinary medicine”, where clinical students reported greater certainty in their attitudes with the median answer “Neutral” as opposed to preclinical students reporting “I disagree” (*p* = 0.017). The rest of the differences in median attitudes between preclinical and clinical students showing no statistical differences are available in the Supplementary File S2.

Analogously to the knowledge part of the questionnaire, possible answers to the attitude statements were dichotomized into desirable and undesirable attitudes. Students presented a highly significant difference between the overall attitudes of all the attendants before and after the symposium. The quantities of both desirable and undesirable answers for all questions regarding the attitudes on antimicrobial use and antimicrobial resistance are shown in Table 6. The list of all desirable and undesirable attitudes can be found in the Supplementary File S1.

## 4. Discussion

To the best of our knowledge, this is the first study on the short-term impact of interventional education on the knowledge and attitudes of veterinary students towards AMR and AMS including the differences between preclinical and clinical students. Differences between these two groups, depending on whether they are in the first or the second half of their studies, were expected since the clinical students have finished their pharmacology course and have begun clinical courses and clinical rotations. This comparison was performed both before and after the symposium. So far, only the efficacy of simulation education as an educational tool for teaching infection prevention and control as part of AMS to veterinary medicine undergraduates has been explored [21].

The present study has shown that in general, students gave a statistically significant higher number of correct answers about AMR and AMS knowledge-related questions in the second and third parts of the questionnaire, as well as desirable attitude-related answers in the fourth part. This shows the effectiveness of educational programs on the knowledge and attitudes regarding AMR and AMS which is consistent with similar interventional studies performed on high school students [17,22], medical undergraduates [23], and veterinary medical undergraduates [21], as well as on healthcare workers [2,18]. 

Regarding the knowledge-related questions from the second section consisting of Likert-based questions, both preclinical and clinical students showed improved knowledge of AMR transfer after the symposium. As discussed in the Materials and Methods Section, median, lower, and upper quartile were used as measures of central tendency and variability, respectively. Because of the small number of possible outcomes of variability, some medians were the same and still statistically significantly different. However, the statistical test detected different underlying distributions of answers which yielded significance. Practically, this means that there were different frequencies of particular answers between groups. Such changes in the distribution of answers with the greater frequency of desirable answers was interpreted as an increase in the certainty for the answer. Statistically significant improvement in knowledge was visible with the questions regarding the presence of the same resistance gene in the different bacterial species and the possibility of interspecies transfer, horizontal transfer of the resistance genes between unrelated bacteria, vertical transfer of the resistance genes, and the role of non-pathogenic environmental bacteria serving as donors of resistance genes. While these methods of antimicrobial gene transfer are well known and documented [24,25], the need for additional education on these topics is obvious, as Australian postgraduate veterinary students showed uncertainty around the risk of AMR transfer between humans and animals [12]. Similarly, the study conducted in Bhutan has shown that 21.9% of animal health workers did not agree that resistant bacteria can be spread between animals and also to humans, while 20.1% did not know how to answer [26].

Interestingly, regarding the statements related to the use of antimicrobials before and after the symposium, no differences were observed in either the group of preclinical students or the group of clinical students. When compared with preclinical students, clinical ones have shown greater certainty in their answers to only three out of 18 knowledge-related questions after the symposium (“Antibiotics are useful in the treatment of viral infections (e.g., influenza)”, “Antibiotics can kill the physiological flora of the body”, and “By killing the body’s physiological flora, antibiotics allow the development of secondary infections”). Although students knew that antibiotics are not useful in the treatment of viral infections even before the symposium, clinical students showed more certainty in their post-education answers when compared to preclinical students. The possible reason why preclinical students showed doubt in their answers may be the fact that they are aware of the use of antibiotics in the management of secondary bacterial infections associated with viral diseases, which is a common practice as seen in the study of Ihedioha et al. [27]. Besides, clinical students have shown higher certainty when answering whether antibiotics could kill the physiological flora of the body, both before and after the symposium. Surprisingly, when asked if by killing the body’s physiological flora, antibiotics allow the development of secondary infections—which is a well-known occurrence, as seen in de Nies et al. [28]—clinical students have shown greater certainty in their affirmative answers only after the symposium, but not before. 

Although it is well established that suboptimal antibiotic concentrations are linked to poor treatment outcomes, can exert non-lethal selective pressure, and may lead to the amplification of resistant bacteria [29,30,31], there is some uncertainty regarding this issue among students before the symposium. Differences in answers disappeared after the symposium between preclinical and clinical students which existed before the symposium for the questions “Small doses of antibiotics in bacteria cannot stimulate the development of resistance” and “The same antibiotic resistance gene can be present in two different bacterial species”.

The results of the present study have shown that both before and after the symposium, all students strongly believed that even if the symptoms of the disease disappeared before the end of the full antibiotic regimen, the antibiotic treatment should not be stopped. Until recently, this belief was widely spread because of the wrong assumption that the risk of AMR development reduces with a longer duration of antimicrobial therapy. According to recent World Health Organization (WHO) proceedings [32], the latest scientific findings show that prolonged antibiotic exposure after the resolution of clinical signs creates additional selective pressure [6]. Even though there is an increasing amount of evidence supporting benefits of shorter antibiotic treatment durations for common infections in human medicine [33,34,35,36,37], there is a lack of comparative clinical trials in veterinary medicine confirming optimal antibiotic treatment durations. Furthermore, since all students showed no significant change in answers to this statement after the symposium, it is obvious that the previous paradigm is still deeply rooted in the veterinary curricula, and similar educations, such as the one in the current study, should put a strong focus on this problem. Since these new paradigms are still not fully accepted and disseminated in the veterinary field and were not the focus of the educational program, this question may cause ambiguity among the students. Therefore, for the evaluation of the educational program in this study, we considered the statement “If the symptoms of the disease disappear before the end of the antibiotic regimen, they can be stopped” as a false one.

The education in the current study also resulted in an overall significant improvement in attitudes regarding AMR and AMS. Even though the distribution of answers before the symposium showed that some clinical students overestimate the knowledge of veterinarians on the right use of antibiotics, after the symposium, clinical students significantly recognized that veterinarians do not know enough about the correct use of antibiotics. This initial attitude may be attributed to the false confidence clinical students obtain throughout their clinical rotations. Furthermore, the Dunning–Kruger effect implies that people without major experience may at earlier stages overestimate their professional abilities and knowledge. The same effect has been discussed in medical trainees [38]. Moreover, since the majority of clinical students were in the fourth or fifth year of their studies, implying that they do not have significant clinical experience, the Dunning–Kruger effect may be one of the reasons for a higher initial estimate of veterinarian knowledge of AMS. A major study on AMS education in European veterinary curricula also observed the positive impact of having completed the clinical rotations on average perception of preparedness in AMS [6]. Gaps in the knowledge and attitudes of both veterinary workers and veterinary students have been observed in Bhutan [26] and the United States [39], as well as in Bangladesh, Serbia, Nigeria, and Poland, respectively [8,9,10,11]. Paradoxically, when assessing the sufficiency of their knowledge about the rational use of antibiotics, both preclinical and clinical students took a conservative stance before and after the symposium, which shows that they are somewhat aware of all the intricate aspects of this problem. This discrepancy, where students at the same time overestimate qualified veterinarians’ knowledge and underestimate their own knowledge, may be due to a lack of experience or exposure, where students are not fully aware of the depth of their knowledge relative to professional standards.

After the symposium in the current study, clinical students have shown greater certainty in agreeing with the statement “Treating animals with the wrong antibiotics will cause AMR in humans”. This again proves that the role of transfer of AMR from animals to humans can sometimes be unclear to veterinary students, which was also demonstrated in the previously referenced study by McClelland et al. [12] where 37.8% of students were not sure whether “resistance to antimicrobials has spread from animals to humans” and 40% percent of them were unsure if “resistance to antimicrobials has spread from humans to animals” when asked about the importance of a certain method of AMR spread. Furthermore, when veterinary students from Belgium were asked for their opinion on the relative contribution of the veterinary use of antimicrobial agents to clinical problems caused by resistant bacteria in humans only 21% of them believed that the impact is high, while 52.4% answered medium [40]. The reason could be that AMR transmission between humans and animals (and vice versa) was studied thoroughly in the last twenty years [41,42,43,44] and probably is not highlighted in the veterinary microbiology curricula yet.

A significant improvement in attitudes in the current study was observed in both preclinical and clinical groups of students regarding their familiarity with the ABCD categorization of antibiotics in veterinary medicine and their understanding of the undesirable subtherapeutic use of antibiotics. When asked about the level of perceived contribution to AMR, 37.8% of Australian postgraduate veterinary students claimed that too low a dose of antimicrobial used in treatment had little or no contribution. This shows the need for emphasis on the role of inappropriate dosages in AMR emergence and spread, as a significant proportion of students, regardless of their geographical location, still lack a comprehensive understanding of how improper antibiotic dosages contribute to AMR. Educational interventions should prioritize this aspect to ensure that future veterinary professionals are well educated to combat the rise of AMR through informed and responsible use of antimicrobials. Moreover, out of 14 attitude-related questions, the statistical difference in the median answer between preclinical and clinical students existed for only one question before the symposium, but with no differences after the symposium. The fact that there is no statistical difference between the median responses of preclinical and clinical students to most of the questions in both of these sections demonstrates the benefits of such training regardless of the stage of education. The effectiveness of educational interventions on different levels of education on attitude change is also visible in previously mentioned similar human medicine studies spanning high school students [17,22] and healthcare workers [2,18].

Since AMS education is at the early stages of development in the veterinary curricula and the link of preclinical subjects such as pharmacology and microbiology with the clinical subjects from the advanced years of veterinary studies is insufficient, there is a clear need for such lectures and discussions of clinical cases [6]. The answers to the third section of the questionnaire, which consisted of eleven MCQ questions about students’ specific knowledge of the rational use of antimicrobials in veterinary practice, showed that in the setting of additional education, discussions about specific scenarios and cases result in a significant improvement of knowledge about the latter. For both questions regarding AMR and surgery, before the symposium, only a small number of students chose correct answers, in the case of the question when they were asked about each hour of surgery, the risk of infection increased (43.18%), and in the case of the question in which ASA status an increased risk of infection occurs (15.91%). Since the majority of students attending the symposium (67.05%) were in the second phase of their studies, which implies certain exposure to clinical education, the absolute quantity of incorrect answers to this question shows the alarming lack of knowledge about the relationship between surgery and the risk of infections. Such knowledge is also crucial for proper antimicrobial use. Still, it must be noted that the inclusion of ASA status in veterinary curricula is relatively recent in Serbia and therefore may explain the perceived inability of the students to apply their anesthesiology knowledge in the AMR context. However, since these answers drastically changed after the symposium, the noted increase in correct answers (85.23% and 75.00%, respectively) shows that these additional educations successfully bridged such knowledge gaps. Another recent study has shown that interventional education in the form of simulation exercises for anesthetic skills acquisition results in a higher proficiency level in fundamental anesthesia skills regardless of the previous amount of experience in anesthesia [45]. Based on a small number of correct answers before the symposium, the need for a greater focus in the curricula is evident in the topics of prebiotics and probiotics as tools in antimicrobial drug use reduction, antimicrobial advice ad hoc expert group (AMEG) classification, and AMR emergence relative to routes of exposure. Only 46.59% of the students knew how to correctly place quinolones, third- and fourth-generation cephalosporins, and polymyxins according to the ABCD (AMEG) classification before the symposium. The AMEG ABCD categorization of antimicrobial drugs in veterinary medicine categorizes antimicrobials into four categories, marked “A” to “D”, with the category “A” representing ‘Avoid’, “B” representing ‘Restrict’, “C” representing ‘Caution’, and “D” representing ‘Prudence’ [46]. Since this classification is relatively new, it is obvious that the students still lack the related awareness and knowledge, but they successfully understand the concept when it is presented, resulting in the rise of correct post-educational answers (77.27%) in the current study. Furthermore, a previous study on veterinary students in Serbia and Croatia [10] has shown that students have a good understanding of AMS but have insufficient knowledge of rational antimicrobial use in clinical practice and biosecurity measures. The current study has shown similar results due to antimicrobial use in clinical veterinary practice. Initially, 61.36% of the students chose correct answers when deciding on antibiotics that have a harmful effect when used in pregnant bitches. However, after the symposium, 87.50% of the students provided the correct answers. This indicates that targeted education can significantly improve students’ knowledge and decision-making skills in this critical area. The results of the current study have shown that scientific workshops and educational programs such as the one in this study could significantly improve the mentioned knowledge gaps. Moreover, these findings underscore the importance of continuous education and training programs focused on practical aspects of antimicrobial use and biosecurity measures. Such programs are essential for equipping future veterinarians with the necessary skills to make informed decisions, thereby reducing the risk of AMR and ensuring better health outcomes for both animals and humans.

This study observes the effect of educational programs on the knowledge and attitudes of veterinary students on AMS. Since the study included preclinical students from the preclinical training phase, it was impossible to include the effect of the education on the practices of the questioned veterinary students. Therefore, there is a need for an additional study on senior veterinary students only with an additional part of the evaluation including student practices. Further studies should be carried out at the level of first-year students and veterinary technicians. An additional limitation of the sampling is the fact that the students who volunteered for the study and showed interest for additional education may by default have better knowledge and understanding of these topics than their peers, so our findings may not necessarily fully reflect the characteristics of all veterinary students in Serbia. Another limitation of the study is in regard to the retention of knowledge. Since the effect was measured only immediately following the educational program, and no follow-up exam was conducted after a longer time after education, it was impossible to evaluate the long-term retention of knowledge and desired attitudes. This was omitted due to the complete anonymity of the attendees, which made it impossible to ensure that the same students would participate in a follow-up exam. Anonymity also limited detailed interpretations of effectiveness on the level of a single student. Since this study observed the effect of education programs only on Serbian students from a single university, a similar study should also be performed encompassing students from different universities and possibly countries.

## 5. Conclusions

Globally, veterinary curricula often fail to adequately inform students about AMR and AMS, resulting in insufficient knowledge and attitudes on these issues. This study demonstrated that additional education, through expert lectures and workshops, effectively bridges these gaps and positively influences students’ understanding and attitudes toward AMR and AMS. Educational interventions significantly improve both general knowledge about AMR and AMS and specific clinical scenarios. The study showed that such education benefits all veterinary students, regardless of their stage in education. Preclinical and clinical students alike demonstrated improved understanding and attitudes post-intervention, with clinical students showing greater confidence due to advanced clinical exposure. Despite these improvements, persistent outdated beliefs highlight ongoing curriculum gaps. Continuous and focused educational efforts are needed to address misconceptions and reinforce critical knowledge areas, especially regarding rational antimicrobial use. While the study demonstrates a need for improved educational integration, it primarily assesses the short-term educational impact rather than long-term professional development. The limitations of this study, including the inability to assess long-term knowledge retention and practical application among students, suggest areas for further research. Future studies should encompass a broader demographic of students from various institutions and potentially different countries, in order to validate and extend these findings. In conclusion, interventional education significantly enhances veterinary students’ understanding and attitudes towards AMR and AMS. Continuous improvement and adaptation of veterinary curricula are essential to equip future veterinarians with the necessary knowledge and attitudes to combat antimicrobial resistance effectively.

## Figures and Tables

**Table 1:** Characteristics of the respondents.

Characteristics	Total Sample		
	n (%)		
Year of Study			
2nd	9 (10.23)	1st phase of studies	29 (32.95)
3rd	20 (22.72)		
4th	27 (30.69)	2nd phase of studies	59 (67.05)
5th	20 (22.72)		
6th	12 (13.64)		
Sex			
Male	23 (26.14)		
Female	65 (73.86)		
Main Area of Interest			
Small companion animals	31 (35.23)		
Farm animals and horses	11 (12.50)		
Public health, government, industry, research	6 (6.82)		
All of the above	26 (29.55)		
Not decided	14 (15.91)		
Previous Engagement in Similar Activities/Conferences			
Once	21 (23.86)		
More than once	13 (14.77)		
Never	54 (61.36)		

**Table 2:** Differences in answers to questions regarding knowledge before and after the symposium on antimicrobial use and AMR.

	Preclinical Students			Clinical Students		
Question	Before Symposium M (Q1–Q3)	After Symposium M (Q1–Q3)	*p*-Value	Before Symposium M (Q1–Q3)	After Symposium M (Q1–Q3)	*p*-Value
Antibiotics are useful in the treatment of bacterial infections (e.g., leptospirosis)	4 (4–5)	5 (4–5)	0.126	5 (4–5)	5 (4–5)	0.140
Antibiotics are useful in the treatment of viral infections (e.g., influenza)	2 (1–3)	2 (2–4)	0.283	2 (1–3)	2 (1–2)	0.091
Antibiotics are indicated to reduce any pain and inflammation	2 (2–3)	2 (1–3)	0.359	2 (1–3)	2 (1–2)	0.154
Antibiotics can kill the physiological flora of the body	4 (4–5)	4 (4–5)	0.450	5 (4–5)	5 (4–5)	0.089
By killing the body’s physiological flora, antibiotics allow the development of secondary infections	4 (3–5)	4 (4–5)	0.523	4 (4–5)	5 (4–5)	0.146
Antibiotics can cause allergic reactions	5 (4–5)	5 (4–5)	0.492	5 (4–5)	5 (4–5)	0.221
Inadequate use of antibiotics can lead to the loss of sensitivity of a specific pathogen to the antibiotic	5 (5–5)	5 (5–5)	0.690	5 (4–5)	5 (4–5)	0.866
If the symptoms of the disease disappear before the end of the antibiotic regimen, they can be stopped	2 (1–2)	1 (1–2)	0.112	1 (1–2)	1 (1–2)	0.071
Subtherapeutic use of antibiotics is justified and allowed in some cases	3 (2–3)	2 (1–3)	0.095	3 (2–3)	2 (2–3)	0.134
Resistance cannot be transferred between different bacterial species	3 (2–4)	2 (1–2)	0.008 *	2 (2–3)	2 (1–2)	<0.001 *
Small doses of antibiotics in bacteria cannot stimulate the development of resistance	2 (2–3)	2 (1–3)	0.289	2 (1–2)	1 (1–2)	0.349
Regular use of antibiotics can prevent the spread of resistant bacterial isolates	2 (2–2)	1 (1–2)	0.004 *	2 (1–2)	2 (1–2)	0.481
Vertical gene transfer in bacteria does not affect the spread of resistance	2 (2–3)	1 (1–2)	0.032 *	2 (1–3)	2 (1–2)	0.087
Environmental bacteria that are not pathogenic to animals can serve as donors of resistance genes	4 (3–4)	4 (4–5)	0.002 *	3 (3–4)	4 (3–5)	0.001 *
With frequent use of a certain antibiotic, the animal may acquire resistance to it	4 (4–5)	4 (4–5)	0.952	4 (2–5)	4 (3–5)	0.130
Horizontal transfer of resistance genes can take place between two unrelated bacteria	3 (3–4)	4 (4–5)	<0.001 *	3 (3–3)	4 (3–5)	<0.001 *
Bacterial resistance to antimicrobial drugs can arise spontaneously without prior contact with the antibiotic	3 (2–4)	4 (3–5)	0.001 *	3 (2–4)	4 (3–5)	<0.001 *
The same antibiotic resistance gene can be present in two different bacterial species	4 (4–4)	5 (4–5)	0.034 *	3 (3–4)	4 (4–5)	<0.001 *

* There is a statistically significant difference; M = median, Q1 = lower quartile, Q2 = upper quartile; 1 = “I strongly disagree”; 2 = “I disagree”; 3 = “Neutral”; 4 = “I agree”; 5 = “I strongly agree”.

**Table 3:** Multiple-choice questions with correct answers.

No.	Question	Answers
1.	In the event of sudden increased mortality on a chicken farm, the veterinarian should do the following:	Wait for a short period of time for the disease’s clinical picture to manifest, then based on it, establish a diagnosis and administer antibiotic therapy
		Administer broad-spectrum antibiotic therapy without establishing a diagnosis
		Perform a pathological examination of the deceased individuals, take samples for laboratory analysis, wait for the analysis results, and then administer antibiotic therapy *
2.	Applying good biosecurity measures on a poultry farm leads to:	Increased use of antimicrobial drugs on farms
		Decreased use of antimicrobial drugs on farms *
		Biosecurity measures have no impact on the amount of antimicrobials used
3.	With each hour of surgery, the risk of infection increases:	Not relevant—it does not increase
		2 times *
		4 times
		10 times
4.	An increased risk of infections occurs in which ASA status?	ASA I
		ASA II
		ASA III *
		It does not matter
5.	The use of antibiotics in pregnant bitches according to good veterinary practice requires the following:	X-ray and ultrasound diagnostics
		Prenatal screening and diagnosis of chromosomal abnormalities
		Isolation and identification of pathogens and preparation of antibiogram *
6.	Antibiotics that have a harmful effect when used in pregnant bitches are:	Tetracyclines *
		Beta-lactam antibiotics
		Lincosamides
7.	The use of colistin in the prevention of diarrhea after weaning in piglets causes the emergence of antimicrobial resistance:	True *
		False
8.	Oligosaccharides (fibers) and short-chain polysaccharides that are not digested by host animals, but are used by specific populations of intestinal microorganisms, are called:	Probiotics
		Prebiotics *
		Phytobiotics
		Macrobiotics
9.	Quinolones, 3rd- and 4th-generation cephalosporins, and polymyxins belong to which category of antibiotics according to the categorization of the European Medicines Agency?:	Category A (“Avoid”)
		Category B (“Restrict”) *
		Category C (“Caution”)
		Category D (“Prudence”)
10.	The greatest estimated impact on antibiotic resistance is attributed to:	Local individual treatment (e.g., teat injector, eye or ear drops)
		Parenteral individual treatment (intravenous, intramuscular, subcutaneous)
		Oral individual treatment (e.g., tablets, oral bolus) *
11.	Metaphylaxis is:	The use of antibiotics in cases where some animals in the herd are clinically ill, while others are subclinically infected or in the incubation period *
		Local application of antibiotics
		The use of multiple different types of antibiotics
		The use of antibiotics for animal growth promotion purposes

* Correct answer.

**Table 4:** Chi-squared analysis of knowledge related to antimicrobial use and AMR, as well as on rational use of antimicrobials in veterinary practice regarding practical issues.

	Number of Correct Answers (%)	Number of Wrong Answers (%)	Total Number of Answers (%)
Before symposium	1609 (63.05%)	943 (36.95%)	2552 (100%)
After symposium	2127 (83.35%)	425 (16.65%)	2552 (100%)
Chi-squared	*p* < 0.001		

**Table 5:** Differences in answers to questions regarding attitudes before and after the symposium on antimicrobial use and AMR.

	Preclinical Students			Clinical Students		
Questions	Before Symposium M (Q1–Q3)	After Symposium M (Q1–Q3)	*p*-Value	Before Symposium M (Q1–Q3)	After Symposium M (Q1–Q3)	*p*-Value
Antimicrobial resistance is a serious problem in my country	5 (4–5)	5 (4–5)	0.468	5 (4–5)	5 (4–5)	0.210
Veterinarians know enough about the correct use of antibiotics	3 (2–3)	2 (2–3)	0.139	3 (2–4)	2 (2–3)	0.028 *
Treating animals with the wrong antibiotics will cause antimicrobial resistance in humans	4 (4–5)	5 (4–5)	0.071	4 (4–5)	5 (4–5)	0.003 *
Antimicrobial resistance is among the most alarming threats to public health	5 (4–5)	5 (4–5)	0.709	4 (4–5)	5 (4–5)	0.052
Broad-spectrum antibiotics are a justified choice for the treatment of all bacterial infections	2 (1–2)	2 (1–2)	0.136	2 (1–3)	2 (1–2)	0.033
Individual effort has negligible impact on antimicrobial resistance	2 (2–3)	2 (2–3)	0.650	2 (1–4)	2 (2–3)	0.812
If the owner requests so, it is okay to give antibiotics to animals without indications	1 (1–2)	1 (1–2)	0.314	1 (1–2)	1 (1–1)	0.117
Veterinarians need additional education to fully understand antimicrobial resistance	5 (4–5)	5 (4–5)	0.483	4 (4–5)	4 (4–5)	0.465
I am familiar with the concept of antimicrobial stewardship	3 (2–3)	4 (3–4)	0.001 *	3 (3–4)	4 (3–4)	0.003 *
Veterinarians should be familiar with the basic characteristics of individual bacterial species in order to avoid inappropriate and ineffective antibiotic therapy	4 (4–5)	5 (4–5)	0.241	4 (4–5)	5 (4–5)	0.057
I consider my knowledge about antimicrobial resistance and the rational use of antibiotics to be sufficient	2 (2–2)	2 (2–3)	0.020 *	2 (1–3)	3 (2–3)	<0.001 *
When choosing antibiotics, it is justified to take the owner’s preferences into account	1 (1–2)	1 (1–2)	0.811	1 (1–3)	1 (1–2)	0.120
I am familiar with the ABCD categorization of antibiotics in veterinary medicine	2 (2–2)	4 (3–4)	<0.001*	3 (2–3)	4 (3–5)	<0.001 *
I always find the subtherapeutic use of antibiotics undesirable	3 (3–3)	5 (4–5)	<0.001*	3 (2–4)	4 (3–5)	<0.001 *

* There is a statistically significant difference; M = median, Q1 = lower quartile, Q2 = upper quartile; 1 = “I strongly disagree”; 2 = “I disagree”; 3 = “Neutral”; 4 = “I agree”; 5 = “I strongly agree”.

**Table 6:** Chi-squared analysis of attitude-related questions on antimicrobial use and AMR.

	Number of Desirable Answers (%)	Number of Undesirable Answers (%)	Total Number of Answers (%)
Before symposium	805 (65.34)	427 (34.66)	1232 (100)
After symposium	984 (79.87)	248 (20.13)	1232 (100)
Chi-squared	*p* < 0.001		

## Data Availability

The data presented in this study are available on request from the corresponding authors.

## Supplementary Materials

The following supporting information can be downloaded at https://www.mdpi.com/article/10.3390/ani14182736/s1: Supplementary File S1. Questionnaire; and Supplementary File S2.
