# Supplementary material for: The Short-Term Impact of Educational Programs on Knowledge and Attitudes Regarding Antimicrobial Stewardship among Veterinary Students in Serbia
Source: Animals (Basel). 2024 Sep 21;14(18):2736. doi: 10.3390/ani14182736 (PMC11428580; doi:10.3390/ani14182736)
Supplement: Supplementary file 1 [file animals-14-02736-s001.zip › animals-3197692-supplementary.pdf]

Supplementary File S1

QUESTIONNAIRE ON KNOWLEDGE AND ATTITUDES OF STUDENTS ABOUT RATIONAL USE OF ANTIBIOTICS

SOCIODEMOGRAPHIC AND ACADEMIC CHARACTERISTICS

Choose the letter in front of your selection.

1. Choose your sex
   a) Male
   b) Female
2. How old are you? (Enter number of years)

   ____

3. What is your main area of interest?
   a) Pets
   b) Horses
   c) Cattle
   d) All of the above
   e) Public health, government, industry, research
   f) I have not decided yet
4. What year of veterinary studies are you presently in?
   a) First
   b) Second
   c) Third
   d) Fourth
   e) Fifth
   f) Sixth
5. In the past, I have already participated in congresses/workshops and similar types of antimicrobial resistance educations
   a) Once
   b) More than once
   c) I have never attended such events

GENERAL KNOWLEDGE REGARDING ANTIMICROBIAL USE AND ANTIMICROBIAL RESISTANCE

In this part of the questionnaire, choose only one answer!

Please enter the symbol X into the box corresponding to your choice.

	I completely disagree	I disagree	Neutral	I agree	I completely agree
Antibiotics are useful in the treatment of bacterial infections (e.g. leptospirosis)	0	0	0	1	1
Antibiotics are useful in the treatment of viral infections (e.g. influenza)	1	1	0	0	0
Antibiotics are indicated to reduce any pain and inflammation	1	1	0	0	0
Antibiotics can kill the physiological flora of the body	0	0	0	1	1
By killing the body's physiological flora, antibiotics allow the development of secondary infections	0	0	0	1	1
Antibiotics can cause allergic reactions	0	0	0	1	1
Inadequate use of antibiotics can lead to the loss of sensitivity of a specific pathogen to the antibiotic	0	0	0	1	1
If the symptoms of the disease disappear before the end of the antibiotic regimen, they can be stopped	1	1	0	0	0
Resistance cannot be transferred between different bacterial species	1	1	0	0	0
Small doses of antibiotics in bacteria cannot stimulate the development of resistance	1	1	0	0	0
Regular use of antibiotics can prevent the spread of resistant bacterial isolates	1	1	0	0	0
Vertical gene transfer in	1	1	0	0	0

bacteria does not affect the spread of resistance					
Environmental bacteria that are not pathogenic to animals can serve as donors of resistance genes	0	0	0	1	1
With frequent use of a certain antibiotic, the animal may acquire resistance to it	0	0	0	1	1
Horizontal transfer of resistance genes can take place between two unrelated bacteria	0	0	0	1	1
Bacterial resistance to antimicrobial drugs can arise spontaneously without prior contact with the antibiotic	0	0	0	1	1
The same antibiotic resistance gene can be present in two different bacterial species	0	0	0	1	1
Subtherapeutic use of antibiotics is justified and allowed in some cases	1	1	0	0	0
**1 = desirable answer**<br>**0 = undesirable answer**					

SPECIFIC KNOWLEDGE ON RATIONAL USE OF ANTIBIOTIC IN VETERINARY PRACTICE REGARDING PRACTICAL ISSUES

In this part of the questionnaire, choose only one answer!

Please circle the letter in front of your choice.

	No.	Question	Answers
	1.	**In the event of sudden increased mortality on a chicken farm, the veterinarian should do the following:**	Wait for a short period of time for the disease's clinical picture to manifest, then based on it, establish a diagnosis and administer antibiotic therapy
			Administer broad-spectrum antibiotic therapy without establishing a diagnosis
			**Perform a pathological examination of the deceased individuals, take samples for laboratory analysis, wait for the analysis results, and then administer antibiotic therapy ***
	2.	**Applying good biosecurity measures on a poultry farm leads to:**	Increased use of antimicrobial drugs on farms
			**Decreased use of antimicrobial drugs on farms ***
			Biosecurity measures have no impact on the amount of antimicrobials used
	3.	**With each hour of surgery, the risk of infection increases:**	Not relevant—it does not increase
			**2 times ***
			4 times
			10 times
	4.	**An increased risk of infections occurs in which ASA status?**	ASA I
			ASA II
			**ASA III ***
			It does not matter
	5.	**The use of antibiotics in pregnant bitches according to good veterinary practice requires the following:**	X-ray and ultrasound diagnostics
			Prenatal screening and diagnosis of chromosomal abnormalities
			**Isolation and identification of pathogens and preparation of**

			**antibiogram** *
	**6.**	**Antibiotics that have a harmful effect when used in pregnant bitches are:**	**Tetracyclines** *
			Beta-lactam antibiotics
			Lincosamides
	**7.**	**The use of colistin in the prevention of diarrhea after weaning in piglets causes the emergence of antimicrobial resistance:**	**True** *
			False
	**8.**	**Oligosaccharides (fibers) and short-chain polysaccharides that are not digested by host animals, but are used by specific populations of intestinal microorganisms, are called:**	Probiotics
			**Prebiotics** *
			Phytobiotics
			Macrobiotics
	**9.**	**Quinolones, 3rd- and 4th-generation cephalosporins, and polymyxins belong to which category of antibiotics according to the categorization of the European Medicines Agency:**	Category A ("Avoid")
			**Category B ("Restrict")** *
			Category C ("Caution")
			Category D ("Prudence")
	**10.**	**The greatest estimated impact on antibiotic resistance is attributed to:**	Local individual treatment (e.g., teat injector, eye or ear drops)
			Parenteral individual treatment (intravenous, intramuscular, subcutaneous)
			**Oral individual treatment (e.g., tablets, oral bolus)** *
	**11.**	**Metaphylaxis is:**	**The use of antibiotics in cases where some animals in the herd are clinically ill, while others are subclinically infected or in the incubation period** *
			Local application of antibiotics
			The use of multiple different types of antibiotics
			The use of antibiotics for animal growth promotion purposes
			*** Correct answer**

ATTITUDES ON THE USE OF ANTIMICROBIAL DRUGS

In this section of the questionnaire, choose your answers based on your own opinions!

Place the symbol X in the box that indicates your choice.

	I completely disagree	I disagree	Neutral	I agree	I completely agree
Antimicrobial resistance is a serious problem in my country	0	0	0	1	1
Veterinarians know enough about the correct use of antibiotics	1	1	0	0	0
Treating animals with the wrong antibiotics will cause antimicrobial resistance in humans	0	0	0	1	1
Antimicrobial resistance is among the most alarming threats to public health	0	0	0	1	1
Broad-spectrum antibiotics are a justified choice for the treatment of all bacterial infections	1	1	0	0	0
Individual effort has negligible impact on antimicrobial resistance	1	1	0	0	0
If the owner requests so, it is okay to give antibiotics to animals without indications	1	1	0	0	0
Veterinarians need additional education to fully understand antimicrobial resistance	0	0	0	1	1
I am familiar with the concept of antimicrobial stewardship	0	0	0	1	1
Veterinarians should be familiar with the basic characteristics of individual bacterial species in order to avoid inappropriate and ineffective antibiotic therapy	0	0	0	1	1
I consider my knowledge	1	1	0	0	0

about antimicrobial resistance and the rational use of antibiotics to be sufficient					
When choosing antibiotics, it is justified to take the owner's preferences into account	1	1	0	0	0
I am familiar with the ABCD categorization of antibiotics in veterinary medicine	0	0	0	1	1
Subtherapeutic use of antibiotics is never desirable	0	0	0	1	1
**1 = desirable answer**<br>**0 = undesirable answer**					

Supplementary File S2

Table S1. Differences in answers to questions regarding knowledge between preclinical and clinical students on antimicrobial use and AMR.

Question	Before			After		
	Preclinical students M (Q1–Q3)	Clinical students M (Q1–Q3)	*p*-Value	Preclinical students M (Q1–Q3)	Clinical students M (Q1–Q3)	*p*-Value
**Antibiotics are useful in the treatment of bacterial infections (e.g. leptospirosis)**	4 (4–5)	5 (4–5)	0.264	5 (4–5)	5 (4–5)	0.603
**Antibiotics are useful in the treatment of viral infections (e.g. influenza)**	2 (1–3)	2 (1–3)	0.520	2 (2–4)	2 (1–2)	**0.002***
**Antibiotics are indicated to reduce any pain and inflammation**	2 (2–3)	2 (1–3)	0.163	2 (1–3)	2 (1–2)	0.112
**Antibiotics can kill the physiological flora of the body**	4 (4–5)	5 (4–5)	**0.015***	4 (4–5)	5 (4–5)	**0.001***
**By killing the body's physiological flora, antibiotics allow the development of secondary infections**	4 (3–5)	4 (4–5)	0.154	4 (4–5)	5 (4–5)	**0.038***
**Antibiotics can cause allergic reactions**	5 (4–5)	5 (4–5)	0.450	5 (4–5)	5 (4–5)	0.389
**Inadequate use of antibiotics can lead to the loss of sensitivity of a specific pathogen to the antibiotic**	5 (5–5)	5 (4–5)	0.261	5 (5–5)	5 (4–5)	0.570
**If the symptoms of the disease disappear before the end of the antibiotic regimen, they can be stopped**	2 (1–2)	1 (1–2)	0.497	1 (1–2)	1 (1–2)	0.678
**Subtherapeutic use of antibiotics is justified and allowed in some cases**	3 (2–3)	3 (2–3)	0.628	2 (1–3)	2 (2–3)	0.409
**Resistance cannot be transferred between different bacterial species**	3 (2–4)	2 (2–3)	0.261	2 (1–2)	2 (1–2)	0.551
**Small doses of antibiotics in bacteria cannot stimulate the development of resistance**	2 (2–3)	2 (1–2)	**0.013***	2 (1–3)	1 (1–2)	0.309
**Regular use of antibiotics can prevent the spread of resistant bacterial isolates**	2 (2–2)	2 (1–2)	0.414	1 (1–2)	2 (1–2)	0.088
**Vertical gene transfer in bacteria does not affect the spread of resistance**	2 (2–3)	2 (1–3)	0.714	1 (1–2)	2 (1–2)	0.477
**Environmental bacteria that are not pathogenic to animals can serve as donors of resistance genes**	4 (3–4)	3 (3–4)	0.488	4 (4–5)	4 (3–5)	0.650
**With frequent use of a certain antibiotic, the animal may acquire resistance to it**	4 (4–5)	4 (2–5)	0.077	4 (4–5)	4 (3–5)	0.856
**Horizontal transfer of resistance genes can take place between two unrelated bacteria**	3 (3–4)	3 (3–3)	0.883	4 (4–5)	4 (3–5)	0.689

**Bacterial resistance to antimicrobial drugs can arise spontaneously without prior contact with the antibiotic**	3 (2–4)	3 (2–4)	0.982	4 (3–5)	4 (3–5)	0.944
**The same antibiotic resistance gene can be present in two different bacterial species**	4 (4–4)	3 (3–4)	**0.008***	5 (4–5)	4 (4–5)	0.128

*** There is a statistically significant difference**
M = median, Q1 = lower quartile, Q2 = upper quartile
1 = "I strongly disagree"; 2 = "I disagree"; 3 = "Neutral"; 4 = "I agree"; 5 = "I strongly agree"

Table S2. Differences in answers to questions regarding attitudes between preclinical and clinical students on antimicrobial use and AMR

**Questions**	**Before**			**After**		
	**Preclinical students M (Q1–Q3)**	**Clinical students M (Q1–Q3)**	***p*-Value**	**Preclinical students M (Q1–Q3)**	**Clinical students M (Q1–Q3)**	***p*-Value**
**Antimicrobial resistance is a serious problem in my country**	5 (4–5)	5 (4–5)	0.630	5 (4–5)	5 (4–5)	0.769
**Veterinarians know enough about the correct use of antibiotics**	3 (2–3)	3 (2–4)	0.585	2 (2–3)	2 (2–3)	0.754
**Treating animals with the wrong antibiotics will cause antimicrobial resistance in humans**	4 (4–5)	4 (4–5)	0.864	5 (4–5)	5 (4–5)	0.533
**Antimicrobial resistance is among the most alarming threats to public health**	5 (4–5)	4 (4–5)	0.344	5 (4–5)	5 (4–5)	0.958
**Broad-spectrum antibiotics are a justified choice for the treatment of all bacterial infections**	2 (1–2)	2 (1–3)	0.928	2 (1–2)	2 (1–2)	0.916
**Individual effort has negligible impact on antimicrobial resistance**	2 (2–3)	2 (1–4)	0.887	2 (2–3)	2 (2–3)	0.773
**If the owner requests so, it is okay to give antibiotics to animals without indications**	1 (1–2)	1 (1–2)	0.727	1 (1–2)	1 (1–1)	0.553
**Veterinarians need additional education to fully understand antimicrobial resistance**	5 (4–5)	4 (4–5)	0.294	5 (4–5)	4 (4–5)	0.160
**I am familiar with the concept of antimicrobial stewardship**	3 (2–3)	3 (3–4)	0.339	4 (3–4)	4 (3–4)	0.966
**Veterinarians should be familiar with the basic characteristics of individual bacterial species in order to avoid inappropriate and ineffective antibiotic therapy**	4 (4–5)	4 (4–5)	0.190	5 (4–5)	5 (4–5)	0.312
**I consider my knowledge about antimicrobial resistance and the rational use of antibiotics to be sufficient**	2 (2–2)	2 (1–3)	0.753	2 (2–3)	3 (2–3)	0.236
**When choosing antibiotics, it is justified to take the owner's preferences into account**	1 (1–2)	1 (1–3)	0.857	1 (1–2)	1 (1–2)	0.295
**I am familiar with the ABCD categorization of antibiotics in veterinary medicine**	2 (2–2)	3 (2–3)	**0.017***	4 (3–4)	4 (3–5)	0.830
**Subtherapeutic use of antibiotics is never**	3	3	0.649	5	4	0.170

**desirable**	(3–3)	(2–4)		(4–5)	(3–5)	

*** There is a statistically significant difference**

M = median, Q1 = lower quartile, Q2 = upper quartile

1 = “I strongly disagree”; 2 = “I disagree”; 3 = “Neutral”; 4 = “I agree”; 5 = “I strongly agree”
